# Effects of dietary garlic (*Allium sativum*) oil on growth performance, haemato‐biochemical and histopathology of cypermethrin‐intoxicated Nile tilapia (*Oreochromis niloticus*)

**DOI:** 10.1002/vms3.1449

**Published:** 2024-04-06

**Authors:** Mustafa Öz, Burak Evren Inanan, Enes Üstüner, Betül Karagoz, Suat Dikel

**Affiliations:** ^1^ Faculty of Veterinary Medicine Department of Fisheries and Diseases Aksaray University Aksaray Turkey; ^2^ Department of Fisheries and Diseases Graduate School of Health Sciences Aksaray University Aksaray Turkey; ^3^ Faculty of Fisheries Department of Aquaculture Cukurova University Adana Turkey

**Keywords:** blood parameters, cypermethrin, garlic oil, growth performance, histopathology, Nile tilapia

## Abstract

**Background:**

When pesticides are introduced into wetlands by agriculture, fish quickly absorb them through their gills. Pesticides reduce hatchability, impede growth, and antioxidant response, killing fish. Therefore, it's crucial to find effective pesticide mitigation methods for fish.

**Objective:**

In this study, the effects of garlic (*Allium sativum*) oil on the growth, haematology, biochemistry and histopathology parameters of Nile tilapia (*Oreochromis niloticus*) exposed to cypermethrin toxicity were investigated.

**Methods:**

In the research, cypermethrin was added to the water of the experimental groups at a rate of 1:20 of the LC_50_ value, and 1.00% garlic oil was added to the fish feed. Fish with an initial weight of 30.26 ± 0.26 g were fed for 45 days.

**Results:**

At the end of feeding, the final weights were determined as 69.39 ± 0.41 (G1), 61.81 ± 0.65 (G2), 82.25 ± 0.36 (G3), and 75.04 ± 0.68 (G4) grams, respectively. Histopathological examinations revealed serious lesions in the gill, liver, brain, and muscle tissues in the cypermethrin group, whereas these lesions were minimal or absent in the garlic oil group.

**Conclusions:**

Garlic oil supplementation had positive effects on growth, haematology, blood biochemistry, hepatosomatic index and histopathological parameters. These findings suggest that garlic oil is a potential protective agent against cypermethrin toxicity.

## Graphical abstract

This study investigates the effects of garlic and low‐dose cypermethrin on Nile tilapia. It was observed that garlic administration positively influenced blood parameters and offered histopathological protection against the adverse effects potentially induced by cypermethrin exposure. Furthermore, garlic treatment was found to have beneficial effects on the growth parameters of the fish.

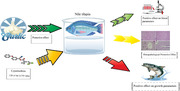

## 1 INTRODUCTION

The delicate balance of aquatic ecosystems is increasingly threatened by human activities, and one of the most significant of them is the use of pesticides (Bashir et al., 2020). They are chemical substances which are specifically designed to control or eradicate pests that adversely affect crop productivity. While their effectiveness in safeguarding agricultural yields cannot be denied, the unintended consequences on nontarget organisms, particularly fish, have raised concerns among scientists and environmentalists alike (Damalas & Eleftherohorinos, 2011). The threat of pesticides to fish is multifaceted as it encompasses various aspects such as acute toxicity, chronic exposure and bioaccumulation. When pesticides enter water bodies, they can exert direct toxic effects on fish, resulting in impaired growth, reproduction and even mortality. Additionally, prolonged exposure to even small amounts of pesticides may cause disruptions of vital physiological processes and compromise the overall fitness and resilience of fish populations (Esbaugh et al., 2018). Moreover, pesticides can bioaccumulate within the tissues of fish. As they move up the food chain, predatory fish such as larger species can accumulate significant levels of these chemicals, posing a threat to both their own health and that of species higher in the chain, including humans who consume contaminated fish (Rohani, 2023).

As a synthetic pyrethroid insecticide, cypermethrin is widely used and causes a danger to aquatic organisms such as Nile tilapia. Its exposure can lead to various harmful effects including compromised growth performance, alterations in haematological and biochemical parameters and histopathological changes. Natural compounds have recently gained attention as potential protective agents against pesticide toxicity (Prusty et al., 2015). The literature provides substantial evidence of the harmful effects of cypermethrin and the protective role of garlic oil. Studies have demonstrated that cypermethrin exposure can lead to neurotoxicity, genotoxicity and teratogenic effects (Assayed et al., 2010a, 2010b; Singh et al., 2012; Wolansky & Harrill, 2008). Furthermore, it has been reported to be harmful to soil bacteria and other microbes (Meena et al., 2020). These findings underscore the wide‐ranging negative impact of cypermethrin on different biological systems.

Nile tilapia (*Oreochromis niloticus*) holds a prominent position in the aquaculture sector, emerging as a vital contributor to global fish production. With its adaptability, rapid growth and versatile culinary attributes, this freshwater species has gained recognition as one of the most economically important and widely farmed species worldwide (Bonham, 2022).

Known for its diverse health benefits, garlic (*Allium sativum*) exhibits promising properties due to its active components such as organosulphur compounds and antioxidants. Active compounds such as allicin and sulphur‐containing compounds in garlic are believed to contribute to its protective effects (Shang et al., 2019). Garlic has historically been utilised in several cultures to combat parasitic, fungal, bacterial and viral infections (Dikel, 2015). Humans have been feeding garlic to animals for millennia and using it to treat many animal ailments (Shalaby et al., 2006).

While there is limited research specifically on the combined effects of garlic oil and cypermethrin in Nile tilapia, studies on garlic and other fish species provide some insights into its potential preventive properties. The present study endeavours to investigate the protective effects of dietary garlic oil on the growth performance, haemato‐biochemical factors and histopathology of Nile tilapia exposed to cypermethrin.

## 2 MATERIALS AND METHODS

### 2.1 Experimental design

The present research was carried out under the ethical permission granted by the ‘Animal Experiments Local Ethics Committee’ on 22 April 2022, in adherence to the guidelines outlined by the Local Ethics Committee. The research used a sample size of 200 Nile tilapia specimens, each having a live weight of 30.26 ± 0.26 g, as the experimental subjects. The fish that was used for our research were selected based on their size. They were organised in evenly distributed groups. In order to determine the amount of cypermethrin to be used in the present study, the method of probit analysis as outlined by Finney (1971) was utilised to find LC50 value for the water. Then cypermethrin was applied at a concentration of 1:20 as the calculated LC50 value in the experiment for a period of 96 h, as stated by Acar et al. (2018). A total of 200 fish was used in this experiment. Cypermethrin was administered at eight distinct ratios as 0.00, 2.00, 4.00, 8.00, 16.00, 32.00, 64.00 and 128 mg/L in the first phase which focused on determining LC50 value while the subsequent phase was dedicated to feeding aspects. Both stages were conducted in laboratory tanks especially designed for animal research purposes. Through the assessment period of 96 h, the fish was monitored three times a day to note LC50 values. Any dead fish were immediately removed from the experimental setup. The 50 L aquariums were aerated during LC50 experiment. Their water was refreshed every day using stocks.

It is important to note that the fish did not receive any food throughout the first phase. It involved conducting three sets of trials with each set consisting of 10 fish residing in 80‐L aquariums. For the second phase of the experiment, groups of 30 fish each were categorised as follows; Group 1 (Control), 0.00% garlic oil in diet, 0.00 mg/L cypermethrine in water; Group 2, 0.00% garlic oil in their diet and Lc50/20 cypermethrine in the water; Group 3, 1.00% garlic oil in their diet and no cypermethrine in the water; and Group 4, 1.00% garlic oil in their diet and Lc50/20 cypermethrine in the water) reaching a total of 120 fish for the second phase. A thermostatic heater of 100 watt manufactured by Eheim was used to maintain a consistent water temperature of 25°C in all experimental groups throughout the study.

### 2.2 Experimental feed preparation and its utilisation in feeding

The fish in the experiment were fed commercial tilapia feed (Hem Yem, Gaziantep, Turkey) with 39% crude protein, 6.7% crude fat, 4.30% crude cellulose and 6.79% crude ash. The experiment used cold‐pressed garlic oil from a commercial source. The fatty acid composition of the garlic oil which was used in the investigation is shown in Table 1. The meals of Groups 3 and 4 in the experiment were supplemented with a mere 1% of garlic oil. The batches of feeds were made in quantities of 100 g each. They were treated with garlic oil which was afterwards mixed with 2 mL of sunflower oil to ensure even dispersion. On the other hand, 3 mL of sunflower oil was introduced into the diets of the other groups of 1 and 2 to maintain uniformity in the feed composition across all groups. The study trial began one day after the first measurement and spanned a duration of 45 days. The fish were provided with two meals every day, one at 08:30 a.m. and another at 4:30 p.m. The experimental protocol included a provision for the fish with a daily diet equivalent to 2% of their body weight.

**TABLE 1:** The fatty acid content of the garlic (*Allium sativum*) oil used in the study.

ID	Compound	Retention time (min)	Concentration (ppm)
1	Octanoic acid, methyl ester	10.649	1.324
2	Decanoic acid methyl ester	15.223	1.899
3	Dodecanoic acid, methyl ester	20.217	2.546
4	Tetradecanoic acid, methyl ester	24.944	7.019
5	Palmitate	29.232	371.391
6	9‐Hexadecenoic acid, methyl ester	30.307	7.311
7	Heptadecanoic acid, methyl ester	31.209	4.627
8	Methyl stearate	33.107	170.471
9	9‐Octadecenoic acid, methyl ester	33.979	851.849
10	9,12‐Octadecadienoic acid, methyl ester	35.379	1694.130
11	Eicosanoic acid, methyl ester	36.625	13.348
12	9,12,15‐Octadecatrienoic acid, methyl ester	36.972	10.285
13	cis‐11‐Eicosenoic acid, methyl ester	37.414	7.594
14	Docosanoic acid, methyl ester	39.885	21.047

### 2.3 Analysing parameters associated with fish growth

The study included the collection of initial (IW) and final weight (FW) measurements using a KERR brand precision balance that had a sensitivity of 0.1 g. The present study included several computations to assess the growth performance and feed data in the experimental setup with following equations:

SpecificGrowthRate(SGR)=[(lnFW−lnIW)/numberofdays]×100, (Company et al., 1999).
Liveweightgain(LWG,g)=FinalWeight(FW)−StartingWeight(IW),
Dailyfeedintake(DFI)=amountoffeedconsumed/time,
Thefeedconversionratio(FCR)=Amountoffeedconsumed/weightgain (Santinha et al., 1999),
Proteinefficiencyratio(PER)=Liveweightgain(g)/proteinintake(g), (Skalli & Robin, 2004),
$\mathrm{The}\mathrm{condition}\mathrm{factor}\left(\mathrm{CF}\right)=\left(\mathrm{Body}\mathrm{weight}\left(g\right)/{\left[\mathrm{Fish}\mathrm{size}(\mathrm{cm})\right]}^{3}\times 100\right)$ (Arellano‐Martínez & Ceballos‐Vázquez, 2001),
Hepatosomaticindex(HSI)(%)=(LiverWeight(g)/BodyWeight(g))×100,
Viscerosomaticindex(VSI)(%)=100×(intestinalweight/bodyweight) (Cheng et al., 2006),
Gonadosomaticindex(GSI)=100(Wg/Wt),

where Wg is the gonad weight and Wt is the total body weight (Amtyaz et al., 2013).

### 2.4 Blood sampling and analysis

Upon completion of the feeding experiment, the fish were subjected to anaesthesia using a concentration of 300 ppm of 2‐phenoxyethanol. Subsequently, the fish were promptly cleaned with a solution of 70% ethanol. Following the cleaning process, blood samples were collected from the vena caudalis using syringes that were pre‐treated with heparin. For haematological analysis, the blood sample was separated into conventional lavender‐top blood collection tubes containing anticoagulant (EDTA). Additionally, standard red‐top (SST™ II) advance serum separator tubes were used to analyse serum biochemical characteristics. The samples underwent centrifugation at a speed of 13,000 × *g* at a temperature of 4°C for a duration of 10 min in order to get serum. The haematological parameters were assessed promptly, while the serums were preserved at a temperature of −80˚C until the biochemical parameters could be analysed. WBCs were counted using a counting chamber. The haematology auto analyser MS4‐S (Melet Schloesing Laboratories, Osny, France) was used to analyse red blood cells (RBCs), mean cell volume (MCV), mean cell haemoglobin (MCH), MCHC, haematocrit (Hct) and haemoglobin (Hb). A manual haematological examination, following the method of Blaxhall and Daisley, was conducted on all blood samples collected in K3EDTA tubes to verify the accuracy of the automated blood count equipment results (Blaxhall & Daisley, 1973). BUN, GLU, ALB, ChoL, CRE, TBL, TPR, GLO and URA were tested. They were measured by Melet Schloesing MScan II biochemical analyser (Osny, France).

### 2.5 Histopathological investigation method

The gills, liver, brain and muscles of Nile tilapia were fixed in 0.1 M phosphate‐buffered formaldehyde solution (pH 7.4). After trimming, the tissues were washed under slow running water for 24 h. Subsequently, they were dehydrated in a series of alcohols (70%, 80%, 90%, 96% and 100%) for varying durations, followed by immersion in xylene and xylene‐paraffin for 30 min each. The tissues were then incubated in soft paraffin (46–48°C) for 15 min and in hard paraffin (56–58°C) for 30 min before embedding in paraffin blocks using Leica EG 1150 H device. Sections of 4 µm thickness were obtained using a Leica RM2125 Rotary Microtome. The sections were stained using the Haematoxylin‐Eosin method and subsequently immersed in alcohol and xylene. The prepared sections were mounted on glass slides using Entellan Merck. The slides were examined under a light microscope (Leica DM‐750), and digital photographs were taken of the areas where lesions were observed (Culling et al., 2014).

### 2.6 Statistical analysis

One‐way ANOVA and post hoc Tukey's HSD tests were performed on the data acquired from each treatment. All statistical studies were conducted with SPSS 18.0 (Illinois, USA). Significant differences occur when *p* < 0.05.

## 3 RESULTS

### 3.1 Determination of LC_50_ value

**FIGURE 1:**
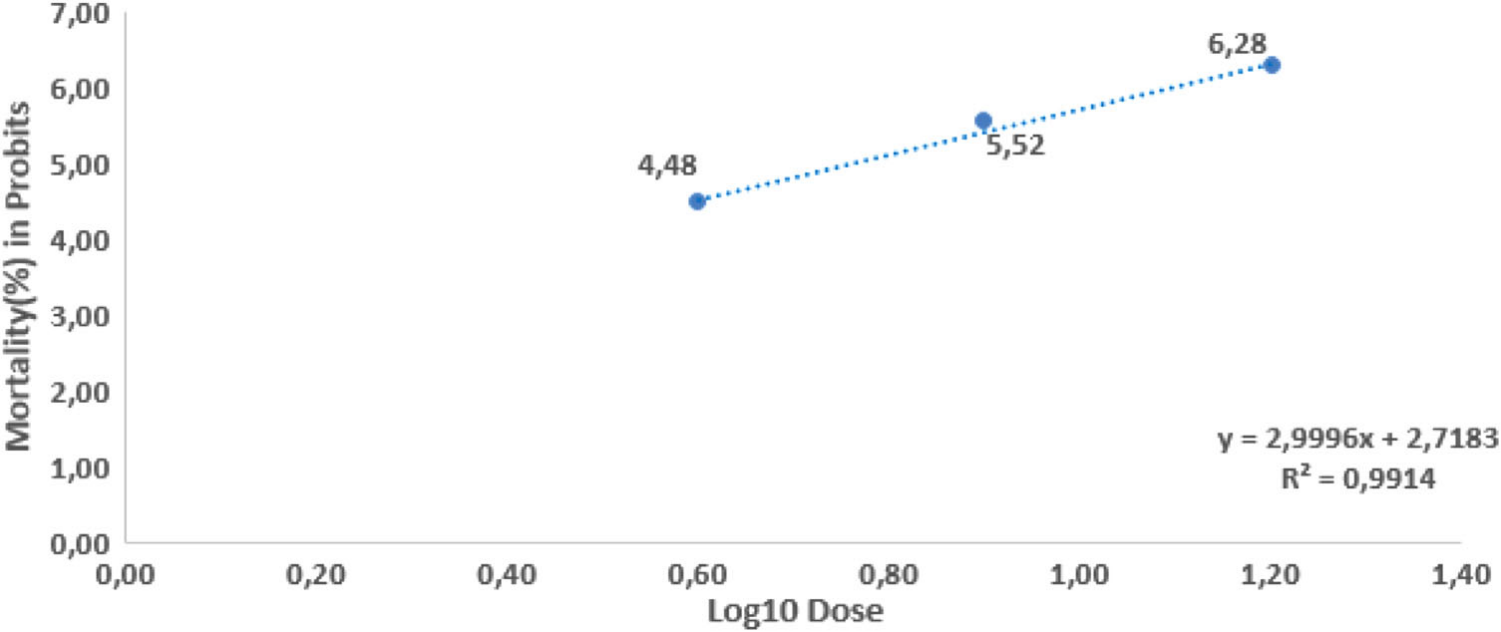
Calculation of 96‐h LC_50_ values of cypermethrin for Nile tilapia (*Oreochromis niloticus)* and regression function with probit.

In the first phase of the study, it was calculated that cypermethrin had a 96 h LC50 of 141.42 mg/L cypermethrin for Nile tilapia. LC50 value of cypermethrin was derived using the regression function from probit analysis (Figure 1), *y* = 2.9996*x* + 2.7183, *R* ^2^ = 0.9914.

### 3.2 Growth parameters

The research examined the effects of adding 1% garlic oil to the diet of Nile tilapia (*Oreochromis niloticus*) which were subjected to cypermethrin (5766/20 mg/L) in water on their development. The findings may be seen in Table 2.

**TABLE 2:** Effect of garlic oil on growth performance, condition factor (CF), gonadosomatic index (GSI), viscerosomatic index (VSI) and hepatosomatic index (HSI) of Nile tilapia against cypermethrin toxicity for 45 days.

	G1 (Control)	G2	G3	G4
**IW**	30.26 ± 0.26	30.26 ± 0.26	30.26±0.26	30.26 ± 0.26
**FW**	69.39 ± 0.41^c^	61.81 ± 0.65^d^	82.25 ± 0.36^a^	75.04 ± 0.68^b^
**FI**	71.96 ± 57^d^	75.04 ± 0.27^c^	79.58 ± 0.17^a^	76.64 ± 0.37^b^
**DFI**	1.60 ± 0.01^d^	1.67 ± 0.01^c^	1.77 ± 0.01^a^	1.70 ± 0.01^b^
**FCR**	1.84 ± 0.01^b^	2.38 ± 0.04^a^	1.53 ± 0.01^d^	1.71 ± 0.03^c^
**SGR**	1.84 ± 0.01^c^	1.59 ± 0.02^d^	2.22 ± 0.01^a^	2.02 ± 0.02^b^
**PER**	1.39 ± 0.01^c^	1.08 ± 0.02^d^	1.68 ± 0.01^a^	1.50 ± 0.02^b^
**CF**	1.62 ± 0.05^b^	1.68 ± 0.06^a^	1.47 ± 0.02^c^	1.61 ± 0.03^b^
**GSI**	1.38 ± 0.01^a^	0.72 ± 0.01^c^	1.31 ± 0.06^a^	1.01 ± 0.07^b^
**VSI**	10.89 ± 0.31^b^	12.06 ± 0.11^a^	10.78 ± 0.14^b^	10.36 ± 0.38^c^
**HSI**	1.89 ± 0.05^b^	2.22 ± 0.06^a^	1.75 ± 0.06^c^	1.85 ± 0.02^b^

*Notes*: G1 (Control): 0.00% garlic oil in diet, 0.00 mg/L cypermethrine in water; G2: 0.00% garlic oil in diet, Lc50/20 cypermethrine in water; G3: 1.00% garlic oil in diet, 0.00 mg/L cypermethrine in water; G4: 1.00% garlic oil in diet, Lc50/20 cypermethrine in water. Each value indicates the average ± standard deviation. The averages expressed using different letters in each row are significantly different (*p* < 0.05).

Abbreviations: IW, initial fish weight; FW, final weight; FI, feed intake; DFI, daily feed intake; FCR, feed conversion rates; SGR, specific growth rate; PER, protein efficiency ratio; CF, condition factor; GSI, gonadosomatic index; VSI, viscerosomatic index; HSI, hepatosomatic index.

### 3.3 Blood parameters

Table 3 presents the haematological data obtained from the investigation. The blood biochemistry of the fish that were included in the study was analysed, and the findings are provided in Table 4.

**TABLE 3:** Protective effect of garlic oil on blood parameters against cypermethrin toxicity in Nile tilapia for 45 days.

	G1 (Control)	G2	G3	G4
**WBC (m/mm^3^)**	4.09 ± 0.06^c^	5.51 ± 0.45^a^	3.41 ± 0.11^d^	4.91 ± 0.15^b^
**RBC (m/mm^3^)**	2.42 ± 0.04^a^	1.72 ± 0.04^d^	2.36 ± 0.03^b^	1.96 ± 0.06^c^
**MCV (fl)**	141.07 ± 3.80^c^	163.322.95^b^	161.50 ± 3.29^b^	184.66 ± 5.11^a^
**MCH (pg)**	33.39 ± 1.15^c^	57.96 ± 2.26^a^	33.17 ± 0.53^c^	43.69 ± 1.76^b^
**MCHC (g/dL)**	23.67 ± 0.35^b^	35.50 ± 1.60^a^	20.54 ± 0.26^c^	23.67 ± 0.84^b^
**Hct (%)**	34.98 ± 2.42^b^	28.08 ± 0.28^c^	38.13 ± 0.35^a^	36.20 ± 0.20^b^

*Notes*: G1 (Control): 0.00% garlic oil in diet, 0.00 mg/L cypermethrine in water; G2: 0.00% garlic oil in diet, Lc50/20 cypermethrine in water; G3: 1.00% garlic oil in diet, 0.00 mg/L cypermethrine in water; G4: 1.00% garlic oil in diet, Lc50/20 cypermethrine in water. Each value indicates the average ± standard deviation. The averages expressed using different letters in each row are significantly different (*p* < 0.05).

Abbreviations: WBC, white blood cell; RBC, red blood cell; MCV, mean cell volume; MCH, mean cell haemoglobin; MCHC, mean cell haemoglobin concentration; Hct, haematocrit.

**TABLE 4:** Protective effect of garlic oil on serum biochemical parameters against cypermethrin toxicity in Nile tilapia for 45 days.

	G1 (control)	G2	G3	G4
**ALP (U/L)**	47.33 ± 1.52^c^	92.00 ± 6.00^a^	42.67 ± 4.72^c^	68.67 ± 2.52^b^
**GOT (U/L)**	39.33 ± 1.53^c^	112.00 ± 1.00^a^	36.00 ± 2.00^d^	85.67 ± 1.53^b^
**GPT (U/L)**	9.67 ± 1.15^d^	31.33 ± 2.52^a^	19.67 ± 1.53^c^	25.67 ± 1.53^b^
**GLU (mg/dL)**	57.66 ± 3.05^c^	95.33 ± 3.79^a^	44.00 ± 1.00^d^	67.67 ± 3.79^b^
**ALB (g/dL)**	0.19 ± 0.01^d^	0.51 ± 0.01^a^	0.30 ± 0.02^c^	0.41 ± 0.01^b^
**CHO (mg/dL)**	51.67 ± 2.51^d^	210.66 ± 1.52^a^	180.67 ± 1.23^c^	144.66 ± 2.52^b^
**CRE (mg/dL)**	0.23 ± 0.01^d^	0.50 ± 0.01^b^	0.34 ± 0.02^c^	0.57 ± 0.06^a^
**TPR (g/dL)**	2.37 ± 0.06^bc^	2.23 ± 0.05^c^	2.73 ± 0.32^a^	2.63 ± 0.05^ab^
**GLO (g/dL)**	2.87 ± 0.06^a^	2.47 ± 0.05^c^	2.93 ± 0.15^a^	2.67 ± 0.06^b^

*Notes*: G1 (Control): 0.00% garlic oil in diet, 0.00 mg/L cypermethrine in water; G2: 0.00% garlic oil in diet, Lc50/20 cypermethrine in water; G3: 1.00% garlic oil in diet, 0.00 mg/L cypermethrine in water; G4: 1.00% garlic oil in diet, Lc50/20 cypermethrine in water. Each value indicates the average ± standard deviation. The averages expressed using different letters in each row are significantly different (*p* < 0.05).

Abbreviations: ALP, serum alkaline phosphatase; GOT, glutamic oxaloacetic transaminase; GPT, glutamic pyruvic transaminase, BUN, blood urea nitrogen, GLU. glucose; ALB, albumin; CHOL, cholesterol; CRE, creatinine; TPR, total protein; GLO, globulin.

### 3.4 Histopathologic findings

**FIGURE 2:**
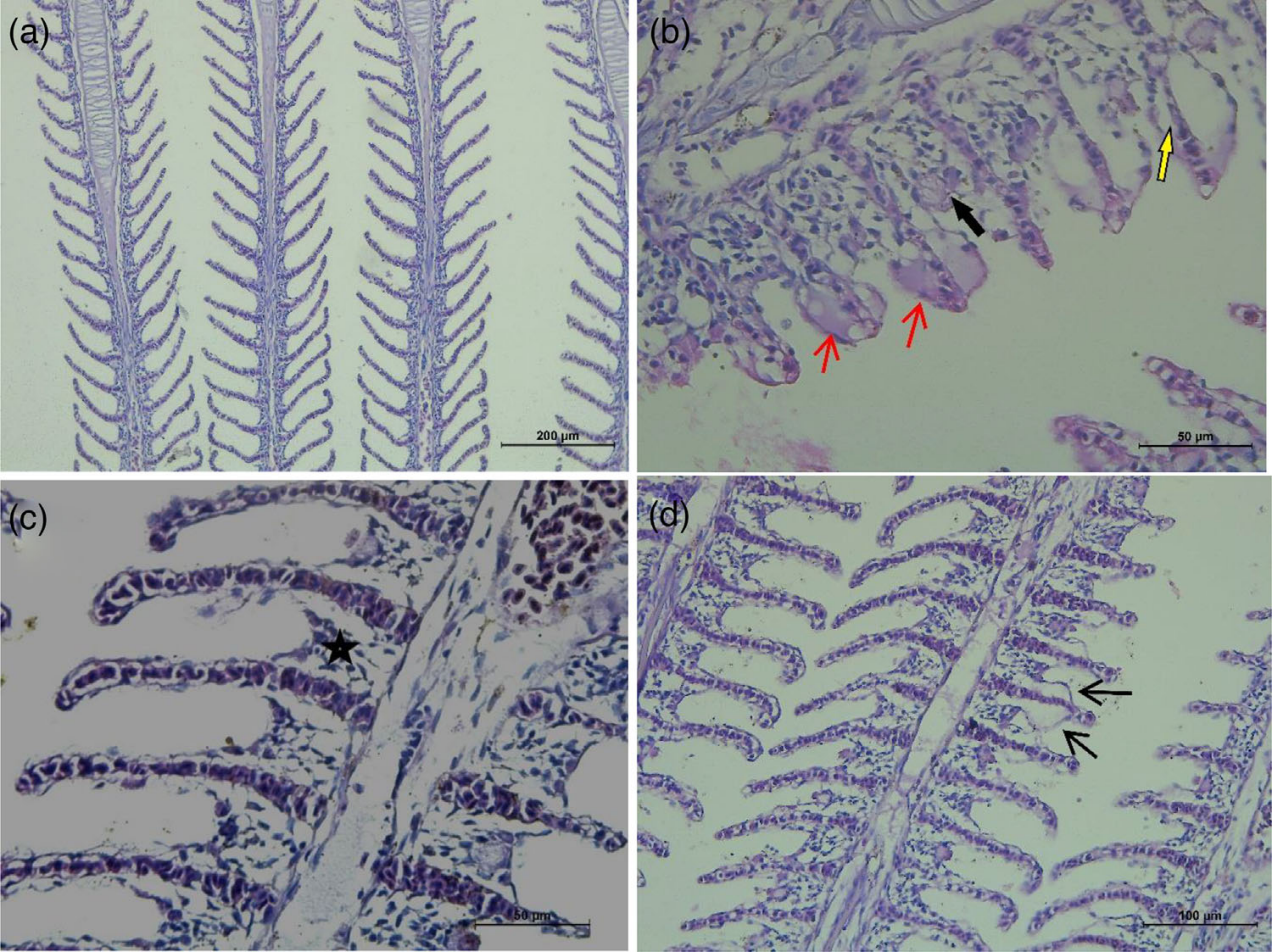
Gill tissue of Nile tilapia (*Oreochromis niloticus*). The normal histological image of (A) Nile tilapia (*O. niloticus*) gills, Group 1 haemotoxylin‐eosin staining (H‐E). (B) +3 Severe lesions on gills. Oedema and desquamation of secondary lamellae (red arrow). Lamellar fusion (black arrow). The separation of epithelial cells of secondary lamellae (yellow arrow), Group 2 (H‐E). (C) +1 Minimal lesions on gills. Lamellar fusion (star), Group 3 (H‐E). (D) +2 Mild lesions on the gills. Desquamation of secondary lamellae (black arrow), Group 4 (H‐E).

As a result of examinations with a light microscope, lesions are observed in the gills, liver, brain and muscles (Table 5) of the fish. The most affected organs are the gills (Figure 2) and liver. In Group 1 (control group), no lesions are observed in the gills, and the primary and secondary lamellae appear histologically normal. In Group 2, desquamation, oedema, hyperplasia and fusion are observed in the secondary lamellae of the gills. Minimal lesions are observed in the primary lamellae and secondary lamellae in Group 3, close to the normal histological appearance of the gills. Compared to Group 2, the gills in Group 4 have minimal lesions and appear close to their normal histological appearance.

**TABLE 5:** Semiquantitative histopathological findings of Nile tilapia after being exposed to Lc50/20 concentration of cypermethrin (CP) and diets with or without *Allium sativum*.

	Pathological lesions	Control	G2	G3	G4
**Gill**	Desquamation of secondary lamellae	0	2–3	0	1–2
	Oedema in secondary lamellae	0	2–3	0	1–2
	Lamellar fusion	0	3	0–1	2
	Epithelial separation	0	3	0–1	2
**Liver**	Vacuolar degeneration	0	3	0	2
	Hydropic degeneration	0	3	0	2
	Congestion	0–1	2–3	0	1–2
**Muscle**	Atrophy	0	3	0	1–2
**Brain**	Hyperaemia	0–1	3	0–1	1–2
	Vacuolisation	0–1	2–3	0	1–2

0: absent; 1: mild; 2: moderate; 3: severe.

**FIGURE 3:**
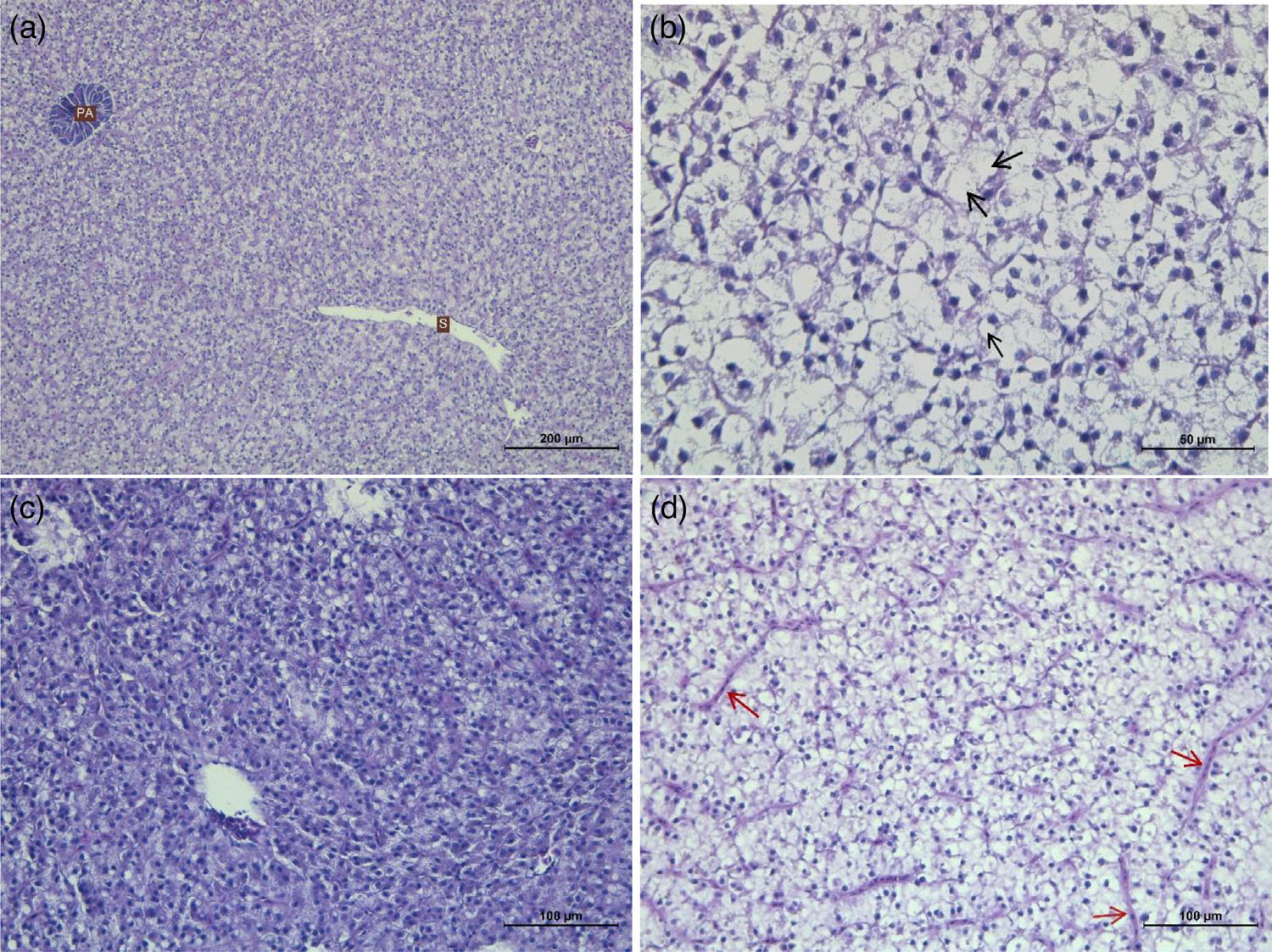
Liver tissue of Nile tilapia (*Oreochromis niloticus*). (A) Nile tilapia (*O. niloticus*) normal histological image of liver, exocrine pancreatic acines (PA), liver sinusoid (S) Group 1 haemotoxylin‐eosin staining (H‐E). (B) +3 Severe lesions in the liver, vacuolar degeneration and hydropic degeneration of hepatocytes (black arrow), Group 2 (H‐E). (C) No liver lesions observed, Group 3 (H‐E). (D) +1 Minimal lesions in the liver, congestion (red arrow), Group 4 (H‐E).

**FIGURE 4:**
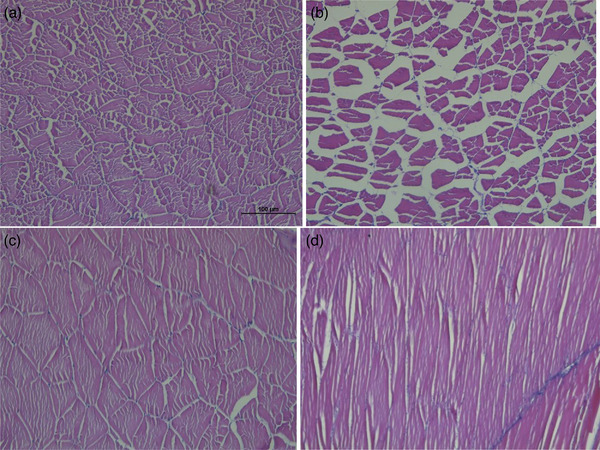
Muscle tissue of Nile tilapia (*Oreochromis niloticus*). The normal histological image of (A) Nile tilapia (*O. niloticus*) dorsal muscle, Group 1 haemotoxylin‐eosin staining (H‐E). (B) +3 Severe lesions in the muscles. Severe atrophy image, Group 2 (H‐E) Bar: 100 µm. (C) No lesions observed in the muscles, Group 3 (H‐E) Bar: 100 µm. (D) +1 Minimal lesions in muscles, Group 4 (H‐E) Bar: 50 µm.

**FIGURE 5:**
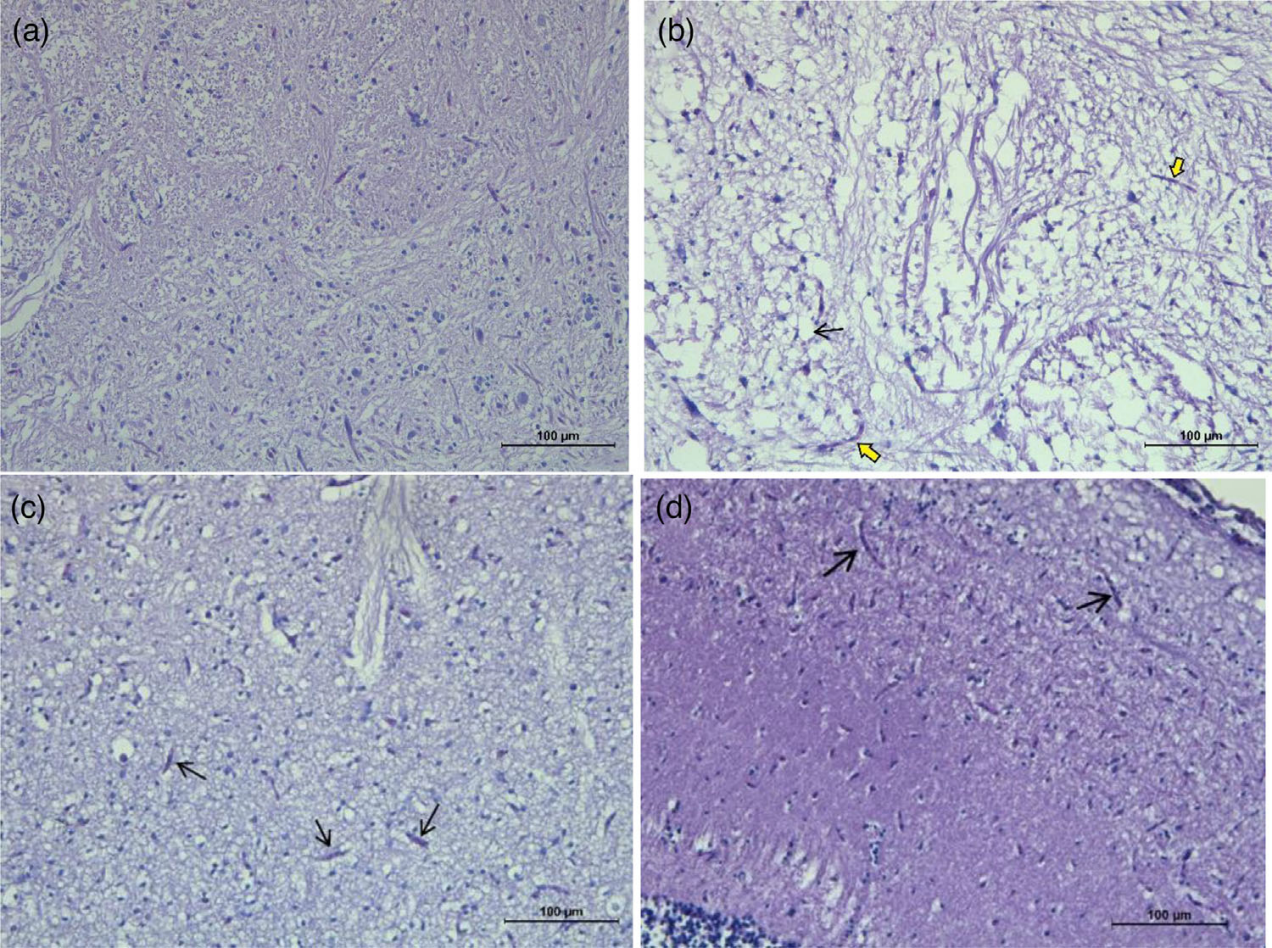
Brain tissue of Nile tilapia (*Oreochromis niloticus*). (A) Nile tilapia (*O. niloticus*) brain normal histological image, Group 1 haemotoxylin‐eosin staining (H‐E). (B) +3 Severe lesions in the brain, vacuolation (black arrow) and hyperaemia (yellow arrow), Group 2 (H‐E). (C, D)  +1 Minimal lesions in the brain, hyperaemia (black arrow), Group 3 and 4 (H‐E).

A normal histological image is seen for liver tissue, exocrine pancreatic acini cells and sinusoidal spaces for Group 1 (Figure 3).  Severe hydropic and vacuolar degeneration, liver sinusoidal spaces, and severe haemorrhages are observed in Group 2. No lesions are seen in Group 3, which has a regular histological image. Minimal lesions are located in Group 4, similar to the normal histological appearance. The muscle tissue is seen as in usual histology in Group 1 (Figure 4). Group 2 depicts atrophy in the muscle tissue. Groups 3 and 4 are found to be compatible with a regular histological appearance. Brain tissue has a normal histological appearance in Group 1 (Figure 5). Intramyelinic oedema and bleeding foci are observed in Group 2. Groups 3 and 4 have regular histological appearance.

## 4 DISCUSSION

The findings which are derived from this investigation demonstrate that the inclusion of garlic oil in fish feed at a concentration of 1.00% exerts a beneficial influence on the growth parameters of Nile tilapia. The study observes that the group of animals who were provided with a feed enriched with garlic oil exhibited the highest growth performance. Conversely, the group of fish that were given water containing cypermethrin at a ratio of Lc50/20 showed the lowest growth performance. Furthermore, the inclusion of garlic oil at a concentration of 1% in the feed of the experimental group, which was exposed to cypermethrin‐contaminated water, exhibited a mitigating effect on the adverse impacts of cypermethrin on growth performance. In the study, garlic oil in fish feed improved the feed conversion ratio (FCR) of Nile tilapia. FCR serves as a crucial metric for assessing the proportion of feed that is effectively transformed into fish meat, thereby representing a significant growth parameter. The study determined that the group of fish fed with garlic oil supplemented feed has the highest computed FCR value (1.53), while the group which was fed with water containing spermetrin had the lowest FCR ratio (2.38). FCR in fish is a critical parameter that directly impacts the efficiency of feed utilisation and, consequently, the economic and environmental sustainability of fish production. Herbal extracts added to fish feed have been shown to have a significant impact on the FCR in various studies. For instance, demonstrated that the extract from dill (*Anethum graveolens*) positively affected the growth performance and body composition of rainbow trout (Sendijani et al., 2020).

Previous research has documented that garlic (*Allium sativum*) possesses various bioactive compounds that exhibit biological properties beneficial for animal health. Notably, ajoene, alliin and allicin are among these compounds which have been found to promote growth and possess antimicrobial, antiviral, antioxidant and antiparasitic properties. Numerous investigations have illustrated the possible application of garlic in bolstering the immunity of fish against aquatic infections (Dikel, 2015; Erguig et al., 2015; Foysal et al., 2019; Shakya & Labh, 2014; Valenzuela‐Gutiérrez et al., 2021). In alignment with the outcomes of the present study, other research has also uncovered that incorporating garlic into diets leads to improved growth performance in rainbow trout (Büyükdeveci et al., 2018; Öz & Dikel, 2022). Previous research has documented that the incorporation of garlic into fish farming practises can enhance fish immune system and promote its growth performance. It can be attributed to the antibacterial, antiparasitic, antioxidant and immunostimulating properties exhibited by garlic (Bender & Bárcenas, 2013; Lee & Gao, 2012; Öz, 2018; Öz & Dikel, 2022). The growth performance of Nile tilapia was found to be positively influenced by the inclusion of garlic in their meals, as observed in another study (Abu‐Elala et al., 2016). Numerous studies have documented the adverse impacts of pesticides on the growth performance of fish, as evidenced by the findings of Dawood et al. (2020), Ko et al. (2019), Murthy et al. (2013) and Rafieepour et al. (2019). A study conducted by Oz et al. (2023) has established that the presence of cypermethrin negatively affects the growth performance of Nile tilapia. Furthermore, Hanson etal. (2007) reported the detrimental effects of three different pesticides on the growth, performance and reproduction of three fish species: *Oreochromis niloticus*, *Chrysichthys nigrodigitatus* and *Clarias gariepinus*. The findings of the study indicate that the use of pesticides impacts on both the development and reproductive abilities of fish.

The gonadosomatic index (GSI) is a measure used to assess the fitness and capacity of fish (Dinh, 2018). Exposure to substances such as pesticides or endocrine disruptors has been shown to have negative effects on reproduction system of fish by reducing their GSI (Arslan et al., 2021; Asifa & Chitra 2019). Numerous studies have demonstrated that fish from contaminated areas exhibit lower GSI compared to those from unpolluted regions (Carvalho Neta & Abreu Silva, 2013). A study, which was conducted in São Marcos Bay, Brazil, discovered that fish living in the area had a decreased GSI compared to fish residing in a clean reference location (Carvalho Neta & Abreu Silva, 2013). Asifa and Chitra (2019) demonstrated a decrease in GSI of cichlid fish, which was exposed to chlordecone. The results in the present study indicate a reduction in GSI within the cypermethrin group. The observed decline in GSI may be ascribed to the disturbances in hormone control and oxidative stress inside the reproductive organs. Nevertheless, it is crucial to consider that the impacts of noxious compounds on gonadal‐somatic index (GSI) might vary based on many aspects such as the particular drug, its concentration, the period of exposure and the species of fish involved.

The assessment of the physiological state and health of fish is a widely used practise in fish research (Parker & Barnes, 2015). The viscerosomatic index (VSI) quantifies the proportional magnitude of the viscera, the internal organs, in relation to the overall body weight of an organism. The present research revealed a rise in VSI among the group administered with cypermethrin. Toxic substances often exert adverse effects on vital metabolic organs, including but not limited to the liver and kidneys. Organ damage can potentially result in the enlargement or increased mass of organs, which could contribute to an elevation in VSI. When VSI is elevated, it suggests that internal organs are growing disproportionately compared to the body size putting strain on these organs. In research concerning fish, scientists use a metric called hepatosomatic index (HSI) to assess the liver weight relative to the total body weight (Murray et al., 2017). According to Murray et al. (2017), an increase in HSI indicates stress levels and a depletion of energy reserves such as glycogen. HSI has proven valuable for evaluating the status of fish as highlighted by Negreiros et al. (2022). Numerous studies have investigated how various chemicals affect HSI of fish. For instance, Murray et al. (2017) states that the presence of nanosilver (nAg) leads to an elevation in HSI index of rainbow trout. A study, which was conducted by Thorpe et al. (2000), demonstrates that the presence of oestrogen compounds such as 17β estradiol and nonylphenol leads to an increase in HSI in the aforementioned species.

Dietary factors may also influence the Homeostasis Model Assessment of Insulin Resistance (HOMA‐IR). An investigation by Ren et al. (2022) demonstrated that the inclusion of taurine supplementation in largemouth bass diet resulted in a decrease in hepatosomatic index (HSI). The observed decrease in HSI among fish, which received taurine supplementation, could be attributed to liver function and reduced stress levels (Ren et al., 2022). In the present study, the comparison of G1 and G2 reveals an increase in HSI within the cypermethrin‐exposed group. Conversely, the comparison of G4 and G2 shows a decrease in HSI value. This observation suggests that garlic has a potentially protective effect against cypermethrin.

The present research aimed to investigate the potential effects of garlic oil supplementation into diets as a means to alleviate the detrimental effects of water‐borne cypermethrin on fish development. It is noted that the inclusion of garlic oil improved growth performance. The findings of the present study indicate that the incorporation of garlic oil into fishmeals holds substantial potential for enhancing the ability of aquaculture industry to mitigate the impact of pesticides. Histopathological examinations are important methods, which are frequently used to detect their effects on fish tissues in laboratory applications (Schwaiger et al., 1996). The gills are one of the most important fish organs that come into contact with water. They serve to clean the blood and conduct respiration similar to the lungs in mammals. Due to these important functions, they are one of the first organs to be affected by a toxic substance. The liver plays a significant role to maintain health in the body by removing toxins and harmful substances (Velmurugan et al., 2009). The histopathology of the liver in fish shows the its general condition and the amount of the effect caused by pollution due to toxic substances (Guilio Richard & Hinton, 2008). It can present structural damage after an interaction with a high concentration of toxic substances (Bruslé & i Anadon, 1996). Korkmaz et al. (2009) reported in their study that cypermethrin causes separation in the gill epithelium, oedema and hypertrophy in epithelial cells, and vacuolar degeneration, congestion, and hypertrophy in hepatocytes in the liver. It has been reported that ascorbic acid given to Nile tilapia fish shows an antagonistic effect and reduces lesions in the gill tissue. Kenthao et al. (2020) investigated acute cypermethrin toxicity in juvenile Nile tilapia fish in their study where they reported desquamation, oedema, hyperplasia, and lamella fusion in the gill tissue, vacuolar degeneration in the liver, increase in sinusoids, and pycnosis. They stated that the severity of the lesions increased when LC50 was increased to LC70. The effect of carbofuran as a pesticide at sublethal concentration in Nile tilapia was examined, and congestion in gill tissue, separation in epithelial layers, separation in secondary lamellae, hypertrophy in hepatocytes in the liver, stasis in sinusoid capillaries, necrosis and cellular fusion were reported (Américo‐Pinheiro et al., 2020). Rajamanickam and Devadason (2021) reported primary and secondary lamella enlargement, destruction, lamellar fusion, epithelial hyperplasia and aneurism in the gills, hypertrophy in hepatocytes, enlargement in cytoplasmic vacuolar and sinusoids, inflammation, vacuolisation and haemorrhage in brain tissue in Nile tilapia exposed to lead poisoning. Ayadi et al. (2015) accounted lamellar epithelial separation, vasodilation, lamellar fusion and filament epithelial proliferation, cytoplasmic vacuolisation in the liver, necrosis and macrophage aggregation in the gills of Nile tilapia exposed to Red 195 in their study. Capkin et al. (2009) reported oedema in secondary lamellae, separation of epithelium, fat droplets in the liver and parenchymal degeneration in the gills of *Oncorhynchus mykiss* exposed to sublethal doses of nitrogen fertilisers. According to Velmurugan et al. (2009), *Clarias gariepinus* experienced cypermethrin lesions in the gills, hypertrophy of the epithelium, hyperplasia of the primary lamellar epithelium, separation of the epithelium, oedema, necrosis and fusion in the secondary lamellae, swelling of the hepatocytes in the liver, focal necrosis and pycnotic nuclei. Rajini et al. (2015) reported diffuse epithelial hyperplasia in the gills, fusion in the secondary lamellae, mononuclear cell infiltration in the primary lamella, congestion in the liver sinusoids, vacuolar degeneration and necrosis in the brain, and cerebellum in their sublethal dose pesticide application on *Danio rerio*.

Soliman et al. (2021) in their study on Nile tilapia found that copper oxide and copper sulphate affected hepatocytes in the liver and resulted in nuclear pycnosis, degeneration in the brain, erosion in the villi, mononucleic cell infiltration in the intestines, renal tubules and degradation in glomerular structure in the kidneys. Jindal and Sharma (2019) reported gliosis, vacuolation, congestion and necrosis in the brain of *Catla catla* fish exposed to cypermethrin. Khafaga et al. (2020) exposed *Cyprinus carpio* to cypermethrin in their study and concluded hepatic tissue deterioration in the liver, vascular congestion, multifocal haemorrhage, diffuse vacuolation areas, fusion in the gills, desquamation, oedema, telangiectasia, tubular occlusion in the tubules and vacuolation in the kidneys as well as atrophy.

The pesticide known as cypermethrin has been shown to have adverse impacts on aquatic species. According to Huang et al. (2016), cypermethrin is a pesticide that specifically acts on molecular components, such as ion channels and ATPase, in fish. Organ dysfunction, including muscle, gills and liver, can be observed due to this disruption in organ functioning. These disturbances in organ function can also lead to changes in blood parameters. A research conducted by Vani et al. (2012) has shown that when fish are exposed to low levels of cypermethrin concentrations, it causes alterations in their haematological parameters. This exposure induces stress and disrupts physiological systems within the fish. Additionally, studies have indicated that fish metabolism may eliminate cypermethrin slower than humans and birds. This difference in elimination rates may explain the increased toxicity of cypermethrin in fish (Saha & Kaviraj 2003). Singh and Zahra (2019) state based on empirical evidence that exposure of fish to cypermethrin can lead to changes in their blood parameters. Like humans, the immune response in fish relies on the participation of white blood cells (WBCs). Fish possess a system consisting of both cellular and humoral mechanisms where immune cells, such as lymphocytes, granulocytes, and monocytes, play vital roles (Milla et al., 2011). In a study conducted by Babu Velmurugan, Senthilkumaar et al. (2016), the number of white blood cells (WBC) increases on the day of exposure to cypermethrin. However, as the concentration of cypermethrin increased, WBC counts decreased after the 21 days. Another research by Majumder and Kaviraj (2017) found that prolonged exposure to cypermethrin concentrations led to anaemia in fish. Additionally, it resulted in reduced growth and a decrease in protein and lipid deposition within their bodies. Piccoli et al. (2019) showed in their study with agricultural workers and their families in Brazil that the use of pesticide may reduce lymphocyte counts and detection of organochlorine pesticides in serum may reduce eosinophil counts. In the present study, an increase in WBC count is observed when G1 is compared with G2. Another decrease in WBC count is seen when G4 is compared with G2. It is assumed to result from the protective effect of garlic. Barathinivas et al. (2022) reported that exposure to pesticides causes a decrease in RBC, Hb and Ht values but an increase in WBC, MCHC, MCV and MCH values in the freshwater catfish *Mystus keletius*. Sweilum (2006) observed that sublethal concentrations of pesticides cause a decrease in erythrocyte count, haematocrit value and haemoglobin content in Nile tilapia (*Oreochromis niloticus*). According to the results of a study by Fathy et al. (2019), exposure of *Oreochromis niloticus* fish to herbicides such as acetochlor, bispyribac‐sodium, bentazon, bensulphuron‐methyl, halosulphuron‐methyl and quinclorac causes changes in the number of red blood cells (RBC), Ht, mean MCV, MCH and MCHC. In the present study, RBC decreased compared to the control group G2. When G4 is compared to G2, an increase in the amount of RBC may be observed. It may suggest that cypermethrin leads to a decrease in erythrocyte production due to the fragmentation of erythrocytes after the increase in ROS (reactive oxygen species), and its effects tissues and organs that play a role in erythropoietin production. Increases in MCV, MCH and MCHC values are observed when G2 is compared with the control group. When G4 and G2 are compared, it is seen that their values are similar to the control group. It is thought that the increase in MCV is due to the larger number of new erythrocytes formed by cypermethrin by breaking down erythrocytes. The increases in MCH and MCHC values occur if the number of RBC decrease but the amount of haemoglobin they contain remains the same. It is because when fewer cells carry the same or increased amount of haemoglobin, the intracellular haemoglobin concentration rises. Comparing the group of garlic supplementation and cypermethrin with the group, which was given only cypermethrin, it is seen that the values are close to the control group, which suggest that garlic may have a protective effect. Hct represents the ratio of the volume of red blood cells to the total blood volume. As a result of the decrease in red blood cell volume, the amount of Hct decreases. Similar results have been reported for *Cyprinus carpio* and *Puntius ticto* (Satyanarayan et al., 2004), *Cyprinus carpio* (Saravanan et al., 2017), *Oreochromis mossambicus* (Ghayyur et al., 2019) and *Oreochromis niloticus* (Dawood et al., 2020).

Fish enzyme parameters such as (ALP), (GOT) and (GPT) provide valuable information about the physiological and biochemical status of fish (Yilmaz, 2020). These parameters can indicate liver damage, oxidative stress and metabolic dysfunction. Enzyme levels for ALP, GOT and GPT are all found to be higher in the G2, which received only cypermethrin. The levels of these enzymes were decreased in G4, to which garlic and cypermethrin were applied, compared with G2. The conclusion that can be drawn from this result is that the damage of cypermethrin to liver can be reduced by the protective effect of garlic. Similar results have been reported for *Rhamdia quelen* (Crestani et al., 2006), *Clarias gariepinus* (Al‐Otaibi et al., 2018), *Oncorhynchus mykiss* (Li et al., 2011) and *Oncorhynchus mykiss* (Ucar et al., 2019). When fish are exposed to toxic substances such as pesticides or pollutants, they can have an impact on their cholesterol levels. Several studies have investigated this phenomenon and observed changes in cholesterol levels in fish as a result. El‐Bouhy et al. (2023) examined the toxicity of a profenofos‐based insecticide on grass carp. The study found that exposure to the insecticide causes a significant decrease in serum cholesterol levels in treated fish compared to control group. Vaseem and Banerjee (2014) conducted a study on biochemical alterations in tissues of *Labeo rohita* after being exposed to the effluent created during extraction of metals from polymetallic sea nodules.

The research on fish so far reveals that the diminished breakdown of cholesterol caused by the hazardous effluent could potentially lead to an elevation in the cholesterol levels observed in fish. In general, the exposure of fish to hazardous chemicals has the potential to induce alterations in cholesterol levels. The specific impacts may vary based on the nature of the harmful chemical and the particular fish species. Potential mechanisms, which cause such alterations, include the suppression of cholesterol production and impairment of androgen signalling. Additional investigation is required in order to fully comprehend the methods and ramifications of altered cholesterol levels in fish that have been subjected to harmful chemicals. The present investigation demonstrates an elevation in cholesterol levels only among the cypermethrin group. A decline was seen in the group where both garlic and the cypermethrin were applied in comparison to the group to which only cypermethrin applied. Exposure of fish to harmful compounds such as bifenthrin or diazinon has resulted in elevated blood glucose levels (Attia & El‐Badawi, 2015; Ghasemzadeh et al., 2015; Mosiichuk et al., 2021; Paunescu et al., 2022; Velisek et al., 2009). The elevation in blood glucose levels is a physiological reaction to the metabolic strain induced by the noxious agent (Attia & El‐Badawi, 2015; Ghasemzadeh et al., 2015; Mosiichuk et al., 2021; Paunescu et al., 2022). Glucose functions as a crucial energy source for essential organs, and fish may enhance glucose production in order to fulfil augmented energy requirements in response to stressful circumstances (Ghasemzadeh et al., 2015; Mosiichuk et al., 2021). The precise processes behind the elevation of blood glucose levels in fish as a result of exposure to hazardous chemicals are not understood completely. Nevertheless, several research has postulated plausible causes. One potential mechanism involves the disturbance of carbohydrate metabolism subsequent to the increase in hepatic glucose activity or glucose synthesis (Yaji et al., 2018). Furthermore, it has been shown that the presence of a hazardous chemical may lead to the production of stress hormones such as cortisol which in turn may have a role in elevating blood glucose levels (Ezemonye & Ikpesu, 2013). The present research has identified an elevation in glucose levels in the group that received only cypermethrin, whereas a reduction in glucose levels is reported in the group that received both cypermethrin and garlic, when compared to the group that received only cypermethrin. This observation suggests that garlic has a possible protective function. The elevation in glucose levels in fish is attributed to its role as an energy source for essential organs and is a consequence of the physiological reaction to the presence of hazardous chemicals. The precise processes that govern this reaction remain unclear and may vary based on the particular hazardous chemical and other influencing variables. Additional investigation is required in order to comprehensively clarify the processes and ramifications associated with elevated glucose levels in fish that have been subjected to hazardous chemicals.

Bioactive compounds found in garlic oil have been reported to possess inhibitory or modulatory effects on the molecular components targeted by cypermethrin. For instance, allicin, the main component of garlic oil, has been demonstrated to exhibit antagonistic effects on ion channels and ATPases affected by cypermethrin (Assayed et al., 2010a, 2010b). Moreover, garlic oil may also have regulatory effects on the endocrine system against hormonal imbalances caused by cypermethrin (Amin et al., 2014).

## 5 CONCLUSIONS

Exposure to cypermethrin has the potential to interfere with several biochemical processes in fish such as oxidative stress, liver function and enzymatic activity. The antioxidant capabilities of garlic have been acknowledged for their potential to mitigate the oxidative damage caused by cypermethrin. Garlic oil supplementation has been shown to enhance antioxidant enzyme activities, reduce lipid peroxidation and maintain the balance of biochemical parameters in fish. Garlic oil significantly enhances fish health by improving growth, feed efficiency and immune responses, while also offering a natural defence against pathogens. Its role in elevating antioxidant enzyme activities and reducing lipid peroxidation underscores its importance in maintaining biochemical balance in fish.

Although garlic oil appears to have some potential to reduce harmful effects of cypermethrin on fish, it is vital to remember that the effectiveness will rely on the precise dose and length of exposure as well as other environmental conditions. Further research is required to acquire more data regarding its protective properties in the particular circumstance concentrating on Nile tilapia and the combination of garlic oil and cypermethrin.

## AUTHOR CONTRIBUTIONS

**Mustafa Öz**: Conceptualization; project administration; supervision; writing – original draft; writing – review and editing. **Burak Evren Inanan**: Data curation; investigation; methodology; writing – review and editing. **Enes Üstüner**: Formal analysis; writing – original draft. **Betül Karagoz**: Formal analysis; investigation. **Suat Dikel**: Formal analysis; investigation; methodology; writing – original draft.

## FUNDING

During the time that this manuscript was being prepared, the authors confirm that they did not receive any funds, grants or other support of any kind.

## CONFLICT OF INTEREST STATEMENT

The authors state unequivocally that they do not have any known financial conflicts of interest or personal relationships that could have given the appearance of influencing the work that is reported in this paper.

## ETHICS STATEMENT

This study was conducted within the framework of ethical approval obtained from ‘Cukurova University Animal Experiments Local Ethics Committee’ on 22.04.2022, in accordance with the principles of the Local Ethics Committee.

## CONSENT TO PARTICIPATE

Every author participated in the design and conception of the study.

## CONSENT TO PUBLISH

The publication of this article was approved by each author.

### PEER REVIEW

The peer review history for this article is available at https://publons.com/publon/10.1002/vms3.1449.

## Data Availability

The data that support the findings of this study are available from the corresponding author upon reasonable request.
